# Surgical outcomes in cervical spondylotic myelopathy with severe cord compression and intramedullary signal changes: a retrospective descriptive study

**DOI:** 10.1007/s00590-026-04832-9

**Published:** 2026-07-01

**Authors:** El Fatih Bashir Elmalik, Laila Metwaly, Mohammed A. A. Mohammed

**Affiliations:** 1 Department of Neurosurgery, Al-Ahli Hospital, Doha, Qatar; 2 Department of Neurosurgery, Riyadh Armed Forces Hospital, Riyadh, Saudi Arabia; 3 Independent Researcher, New York City, USA

**Keywords:** Cervical spondylotic myelopathy, Intramedullary signal changes, Spinal cord compression, Decompression surgery, mJOA score, Retrospective descriptive study

## Abstract

**Purpose:**

To evaluate surgical outcomes in cervical spondylotic myelopathy (CSM) with intramedullary signal changes (IMSCs) and assess the impact of preoperative severity on recovery.

**Methods:**

This retrospective study included 312 patients undergoing cervical decompression. Neurological status was assessed using the mJOA score preoperatively and at 6–12 months. A subset of 43 patients was analyzed separately for inferential statistics using chi-square testing.

**Results:**

Severe CSM was present in 54.2% and moderate in 45.8%. Overall improvement occurred in 53.2%, with a mean mJOA increase of 2.9 points. In the subset, mean mJOA improved from 10.25 (SD 1.80) to 13.16 (SD 2.43). A significant association was found between preoperative severity and outcome (*p* < 0.05).

**Conclusion:**

Surgical decompression leads to meaningful neurological improvement, with outcomes influenced by preoperative severity.

## Introduction

Cervical spondylotic myelopathy (CSM) is the most common cause of spinal cord dysfunction in adults over the age of 50 and represents a leading cause of progressive neurological disability worldwide [1]. Degenerative changes such as intervertebral disc degeneration, osteophyte formation, facet joint arthropathy, and ligamentous hypertrophy may lead to progressive narrowing of the cervical spinal canal and chronic spinal cord compression [1, 2].

Magnetic resonance imaging (MRI) is the primary imaging modality for evaluating CSM. It allows the visualization of spinal cord compression and intramedullary signal changes [3]. T2-weighted intramedullary signal changes are frequently observed in advanced disease and have historically been considered indicators of chronic spinal cord injury and potentially poor surgical prognosis [4, 5]. However, the prognostic significance of these findings remains controversial. Recent studies have also explored quantitative MRI assessment of cervical spinal cord compression and emphasized the clinical heterogeneity of cervical spondylotic myelopathy, highlighting the importance of integrating radiological and neurological evaluation in surgical decision-making [6–8].

Previous studies have shown that neurological recovery may still occur following adequate surgical decompression despite the presence of intramedullary signal changes [1, 9]. Suri et al. reported that these MRI findings may influence surgical outcomes but do not necessarily preclude postoperative improvement [4]. Similarly, Sarkar et al. demonstrated that T2-weighted signal abnormalities may evolve after decompressive surgery, suggesting that they are not always markers of irreversible damage [9]. A meta-analysis by Chen et al. also suggested that preoperative MRI characteristics may have prognostic value but should be interpreted within the broader clinical context [3].

Early surgical intervention plays an important role in preventing further neurological deterioration in patients with CSM [10]. The present study aimed to evaluate surgical outcomes in patients with cervical spondylotic myelopathy presenting with severe spinal cord compression and intramedullary signal changes on MRI. A substantial proportion of patients were treated in Sudan, a lower-resource healthcare setting, providing additional real-world context for the findings. In contrast, a subset of patients was treated in Qatar, which represents a higher-resource healthcare environment with more advanced medical infrastructure.

## Methods

### Study design

This study was designed as a retrospective observational study combining an analytical cohort component with a larger descriptive cohort. The study spans a 25-year period and includes patients treated surgically for cervical spondylotic myelopathy (CSM) by a single senior surgeon in Qatar and Sudan. For transparency, inferential statistical (cohort) analysis was performed on a subset of 43 patients with complete datasets, while the remaining patients were analyzed descriptively due to incomplete data availability.

### Patient population

**Fig. 1:**
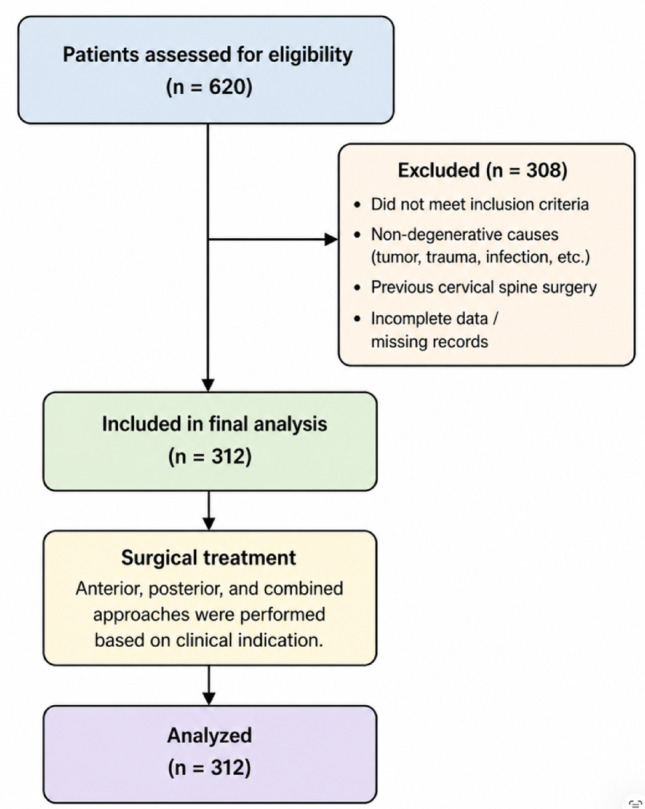
Flow diagram illustrating patient selection for the study. A total of 620 patients who underwent cervical spine surgery were assessed for eligibility. Of these, 308 patients were excluded for not meeting the inclusion criteria, for non-degenerative causes (such as tumor, trauma, or infection), for previous cervical spine surgery, or for incomplete data. The remaining 312 patients met the inclusion criteria and were included in the final analysis. Surgical management included anterior, posterior, and combined approaches based on clinical indication.

A total of 620 cervical spine surgical cases were reviewed. Of these, 312 patients met the inclusion criteria and were included in the final study population. The patient selection process is illustrated in the flowchart in Fig. 1.

Within this population, a subset of 43 patients with complete and well-documented clinical and radiological data was identified. This subset constituted the original analytical cohort, in which detailed statistical analysis was performed. Subsequently, the remaining patients were incorporated to form the full cohort (*n* = 312), which was analyzed descriptively to provide real-world validation and generalizability of the findings.

### Inclusion criteria

Adult patients with clinically confirmed moderate to severe cervical spondylotic myelopathy (mJOA score ≤ 15) were included based on clinical and radiological findings. Eligible patients had degenerative cervical spine pathology, including disc degeneration, ligamentum flavum hypertrophy, facet joint hypertrophy, or ossification of the posterior longitudinal ligament. All included patients demonstrated MRI evidence of severe cervical spinal cord stenosis (sagittal canal diameter < 10 mm) associated with intramedullary signal changes.

### Exclusion criteria

Patients were excluded if they had non-degenerative causes of myelopathy, including tumors, trauma, or demyelinating diseases. Additional exclusion criteria included congenital spinal canal stenosis without degenerative pathology, prior cervical spine surgery, or neurological disorders that could confound functional assessment, such as Parkinson’s disease, stroke, or amyotrophic lateral sclerosis.

### Clinical evaluation

All patients underwent standardized neurological assessment. Clinical manifestations included progressive motor and sensory deficits, hand clumsiness, gait instability, and sphincter dysfunction. Neurological signs included hyperreflexia, Hoffmann’s sign, Babinski’s sign, and Lhermitte’s sign.

Functional status was evaluated using the modified Japanese Orthopaedic Association (mJOA) scoring system (16). Patients were classified according to the Fehlings classification into mild (15–16), moderate (12–14), and severe (< 12) myelopathy [11].

### Radiological assessment

Magnetic resonance imaging (MRI) was performed to confirm cervical spinal cord compression and intramedullary signal changes (IMSCs). Severe cervical cord compression was defined radiologically by marked reduction or obliteration of the cerebrospinal fluid (CSF) space surrounding the spinal cord with evident cord indentation or flattening on MRI, often accompanied by intramedullary T2 signal changes. Imaging was performed using MRI machines of different generations and field strengths (0.5–1.5 Tesla) across centers, resulting in variability in image quality. Consequently, precise subclassification of intramedullary signal changes (e.g., faint/edematous versus sharp/gliotic T2 signal patterns) was not consistently feasible across all patients. Therefore, IMSCs were analyzed as a single entity rather than subdivided into signal intensity subtypes. Consequently, precise subclassification of intramedullary signal changes (e.g., faint/edematous versus sharp/gliotic T2 signal patterns) was not consistently feasible across all patients. Therefore, IMSCs were analyzed as a single entity rather than subdivided into signal intensity subtypes.

### Surgical treatment

All patients underwent surgical decompression based on clinical and radiological indications. The choice of surgical approach (anterior, posterior, or combined) was determined by the location and extent of compression, cervical alignment including preservation or loss of cervical lordosis, number of involved levels, patient’s age, and surgeon preference.

Standard surgical techniques included anterior cervical discectomy and fusion (ACDF), posterior decompression, and combined anterior-posterior approaches where indicated. Posterior decompression was more frequently performed in older patients with multilevel disease, particularly when preservation of sagittal alignment was considered important. ACDF was primarily used in patients with anterior compression involving one to three spinal levels, while combined approaches were reserved for extensive multilevel disease or combined anterior and posterior compression and were performed either during the same operation or as staged procedures.

**Fig. 2:**
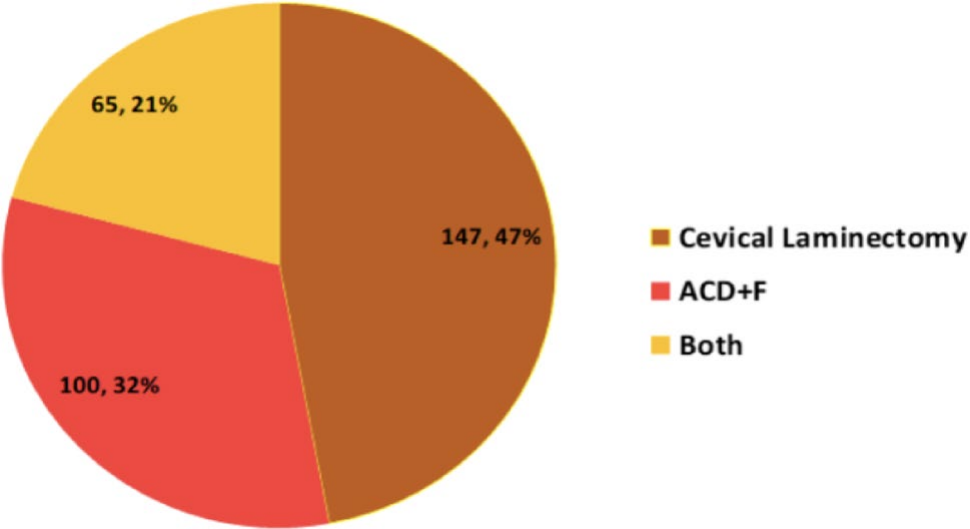
illustrates the distribution of surgical approaches among the 312 patients included in the study. Posterior decompression (cervical laminectomy) was performed in 147 patients (47%), anterior cervical discectomy and fusion (ACDF) in 100 patients (32%), and combined anterior-posterior approaches in 65 patients (21%).

The distribution of surgical approaches among the 312 patients is illustrated in Fig. 2. Posterior decompression (cervical laminectomy) was performed in 147 patients (47%), ACDF in 100 patients (32%), and combined anterior-posterior approaches in 65 patients (21%).

Intraoperative tools included operative microscopy and fluoroscopic guidance. Representative surgical and radiological findings are shown in Fig. 4.

### Follow-up

Patients were followed clinically for 6–12 months postoperatively. Neurological outcomes were reassessed using the mJOA scoring system during follow-up visits.

**Fig. 3:**
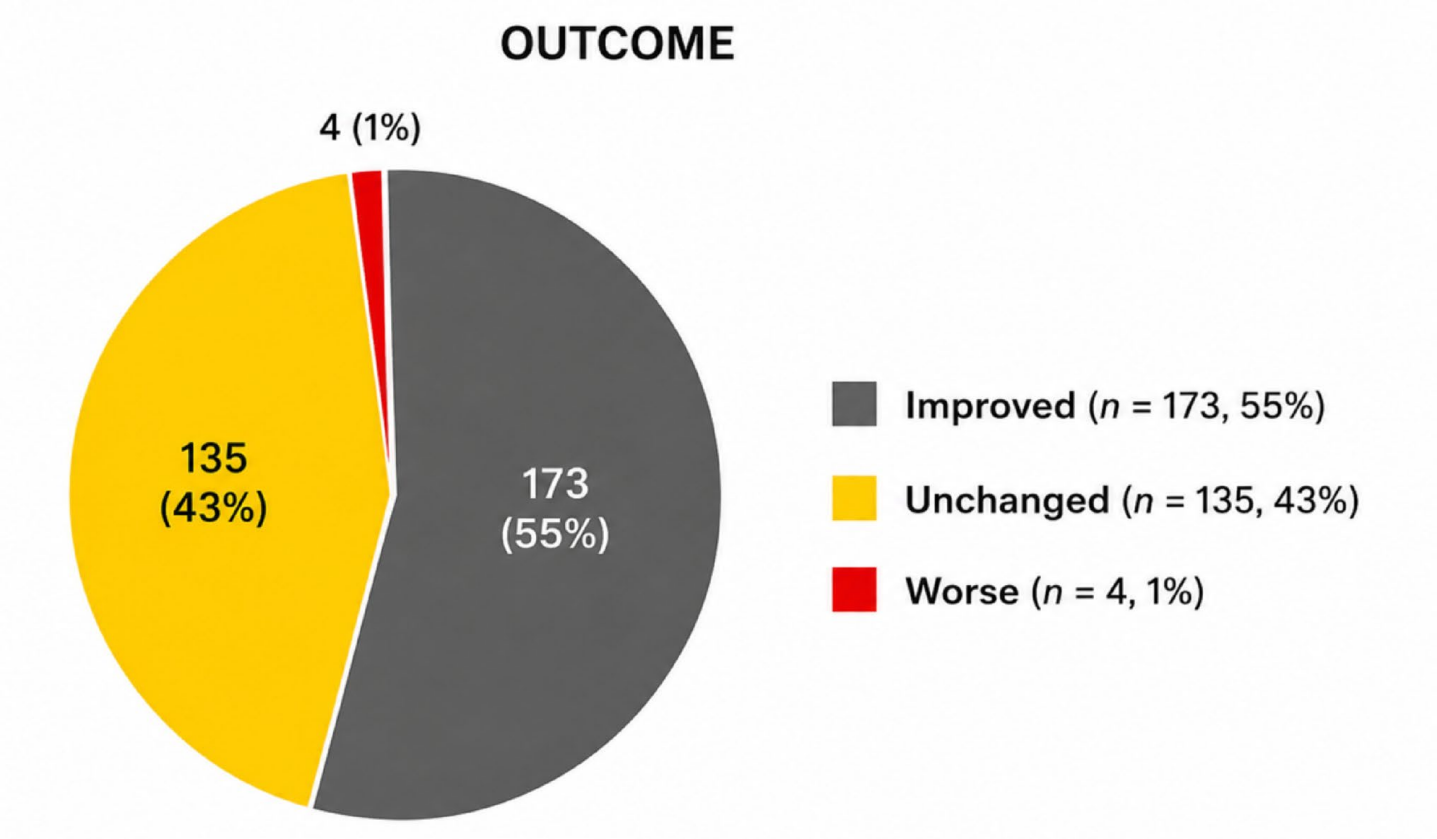
Overall clinical outcomes at final follow-up among the 312 patients included in the study. Neurological improvement was observed in 173 patients (55%), while 135 patients (43%) remained clinically unchanged. Neurological deterioration was observed in 4 patients (1%)

At follow-up, neurological improvement was observed in 173 patients (55%), while 135 patients (43%) remained clinically unchanged. Neurological deterioration was observed in 4 patients (1%). Posterior decompression (laminectomy) was the most commonly performed procedure and demonstrated outcomes comparable to ACDF and combined anterior-posterior approaches. The distribution of outcomes according to surgical approach is summarized in Fig. 3; Table 1.

**Table 1:** Neurological outcomes according to surgical approach

Outcome	Total (312)	ACDF (*n* = 100)	Laminectomy (*n* = 147)	Combined (*n* = 65)
Improved	173	71	68	34
Unchanged	135	28	77	30
Worsened	4	1	2	1
Total	312	100	147	65

### Data presentation and statistical analysis

Descriptive statistical analysis was performed for the full cohort of 312 patients. Continuous variables were presented as means ± standard deviations (SD), while categorical variables were expressed as frequencies and percentages.

Inferential statistical analysis was performed exclusively on the subset of 43 patients with complete datasets, due to data availability. Within this subgroup, continuous variables were reported as means ± SD, and statistical testing using chi-square analysis was applied to evaluate associations between preoperative severity and postoperative outcomes.

The findings from the subset analysis were interpreted in conjunction with the descriptive results of the full cohort, providing complementary statistical insight. A* p*-value < 0.05 was considered statistically significant.

**Fig. 4:**
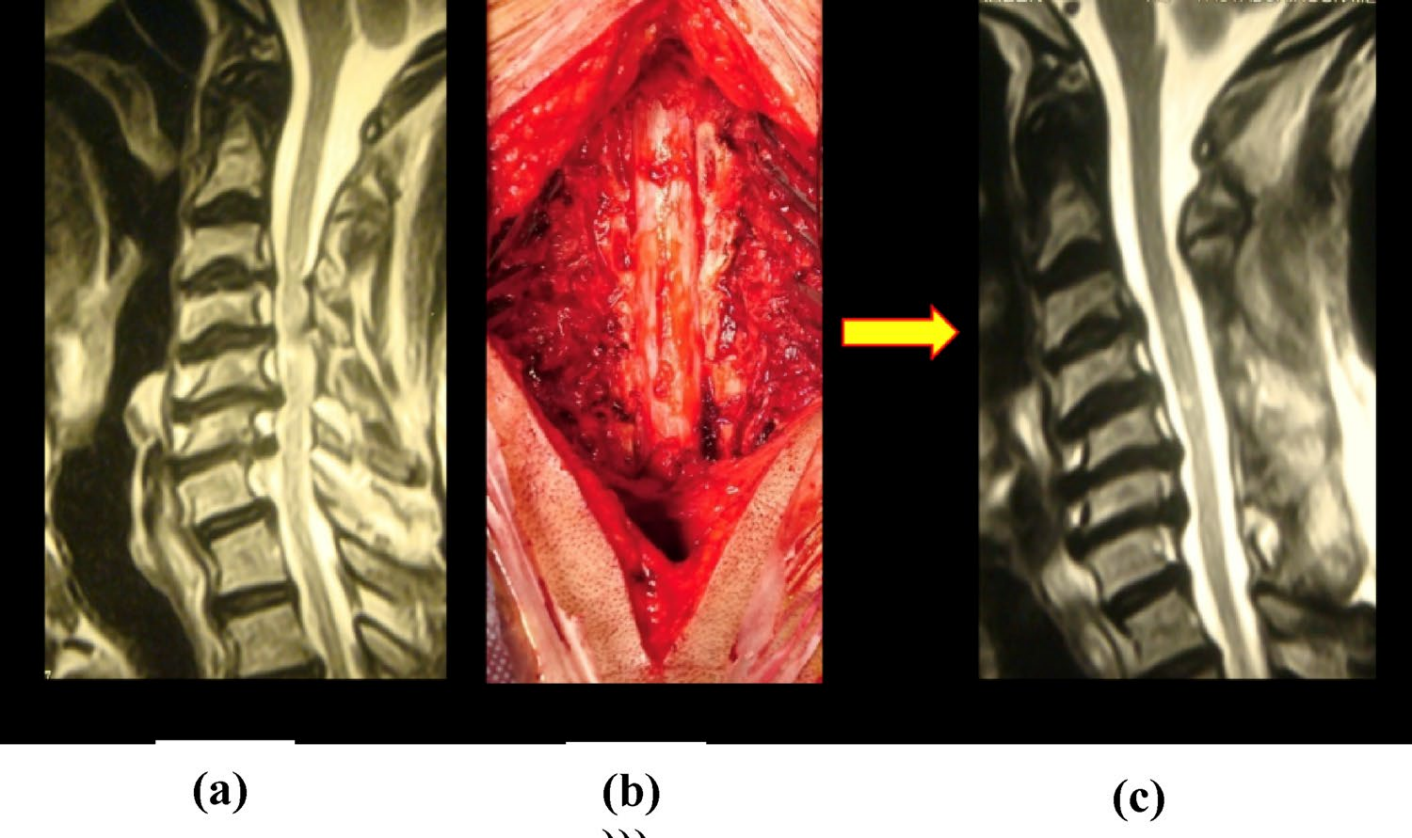
Preoperative sagittal T2-weighted cervical MRI demonstrating severe spinal cord compression with intramedullary signal change **(a)**. Intraoperative photograph showing posterior cervical decompression **(b)**. Postoperative sagittal T2-weighted MRI demonstrating adequate spinal cord decompression following surgery **(c)**

**Fig. 5:**
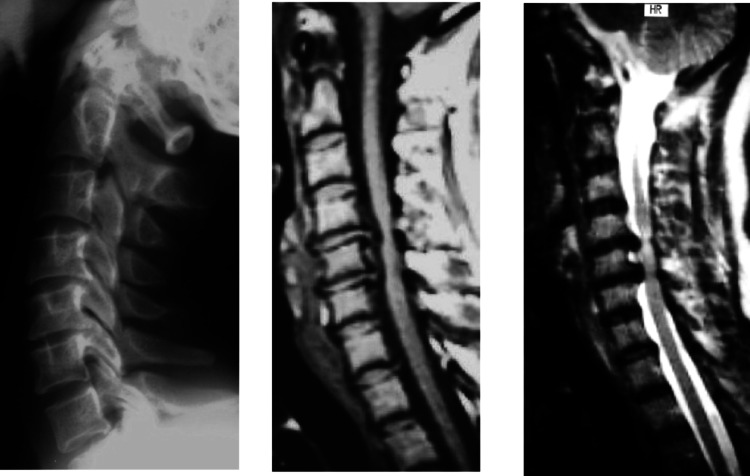
Preoperative lateral cervical spine X-ray showing C4–C5 and C5–C6 narrowed disc spaces with prominent posterior osteophytes **(a)**. Sagittal T1- and T2-weighted MRI images demonstrating a disc/osteophyte complex causing significant spinal cord compression with intramedullary signal changes **(b**,** c)**

**Fig. 6:**
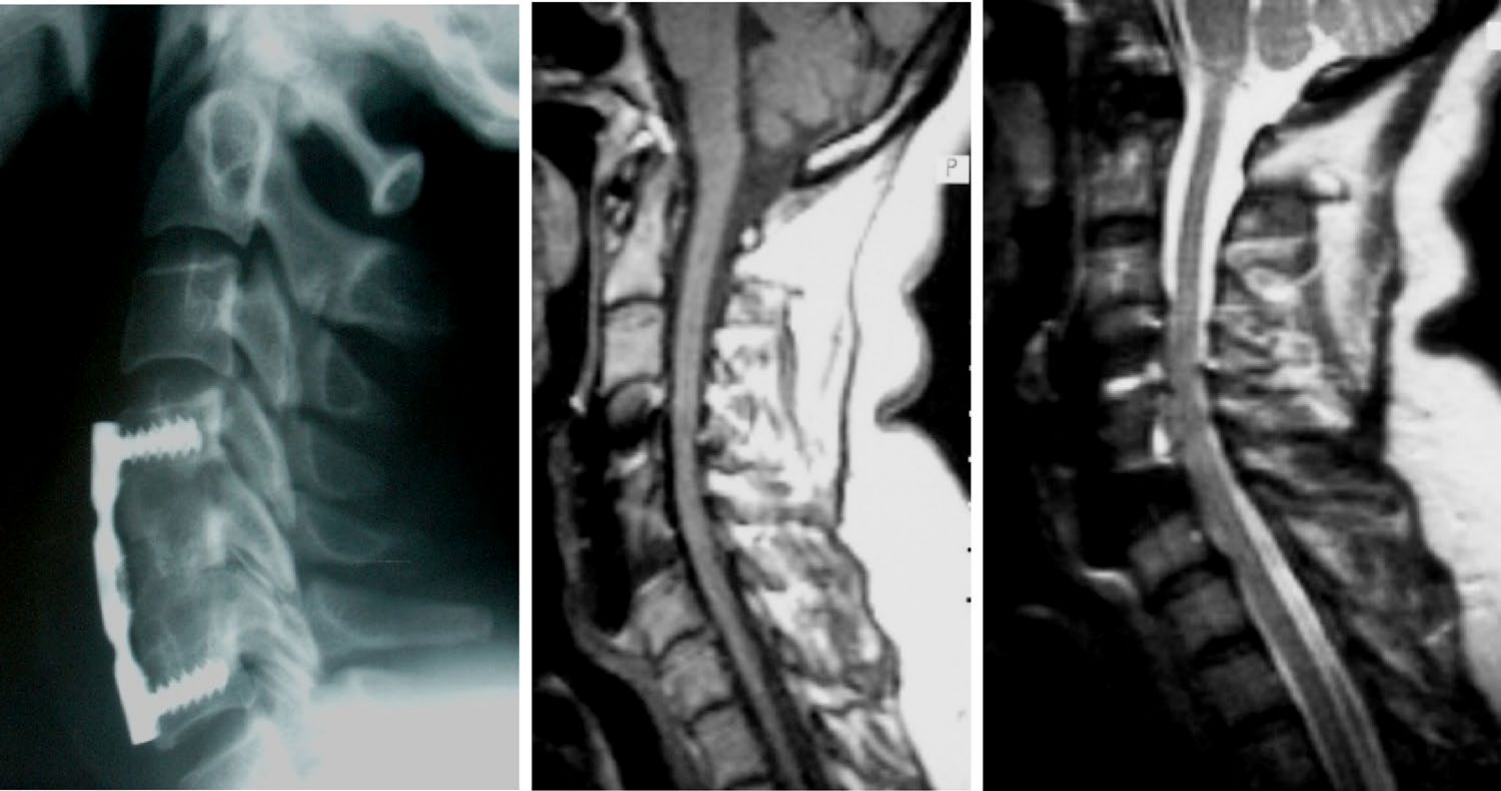
Postoperative lateral cervical spine X-ray showing removal of osteophytes and fusion of the cervical spine from C4 to C6 **(a)**. Postoperative sagittal T1- and T2-weighted MRI images demonstrating evidence of spinal cord decompression **(b**,** c)**

**Fig. 7:**
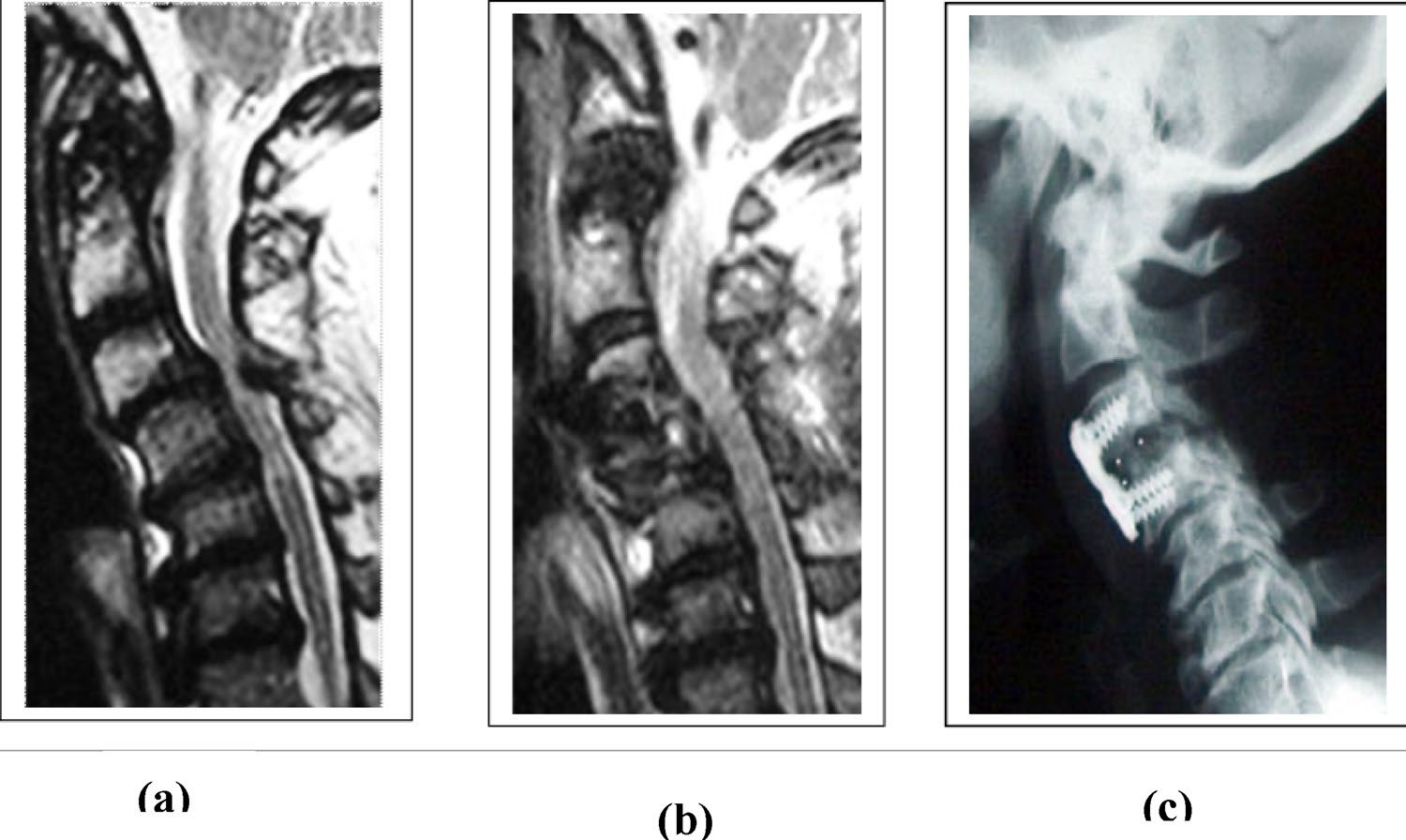
Representative radiological images of cervical spondylotic myelopathy with severe spinal cord compression. Preoperative sagittal T2-weighted MRI demonstrating marked anterior and posterior spinal cord compression at the C3–C4 level with associated intramedullary signal changes **(a)**. Postoperative sagittal T2-weighted MRI following anterior cervical discectomy and fusion + posterior decompressive laminectomy, showing adequate spinal cord decompression **(b)**. Postoperative lateral cervical spine X-ray demonstrating anterior fusion and alignment following instrumented fixation **(c)**

Representative radiological images of CSM cases with cervical spinal cord compression and postoperative decompression are presented in Figs. 4, 5, 6 and 7.

## Results

### Patient demographics

A total of 312 patients were included in the final analysis. The study cohort comprised 201 males and 111 females, with a mean age of 62 years (range 26–83 years). Eight patients were identified as having diabetic peripheral neuropathy. These demographic characteristics of the full descriptive cohort are summarized in Table 2.

In the subset of 43 patients included in the cohort analysis, the mean age was 56 years (SD 12.01), with a median of 55 and a mode of 52. The age ranged from 30 to 80 years (range 50), reflecting a predominantly middle-aged to elderly population.

**Table 2:** Demographic characteristics of the study population

Variable	Value
Total patients	312
Males	201
Females	111
Average age (years)	26–83
Mean age (years)	62
Diabetic peripheral neuropathy	8

### Symptoms duration

In the subset of 43 patients, symptom duration ranged from 2 to 48 months, with a mean of 11.88 months, a median of 6 months, a mode of 3 months, and a standard deviation of 12.29.

### Preoperative severity

According to the mJOA-based severity classification described by Fehlings et al., 169 of 312 patients (54.2%) presented with severe cervical spondylotic myelopathy (CSM), while 143 patients (45.8%) had moderate disease. No patients were preoperatively classified as having mild CSM. A detailed descriptive breakdown of preoperative severity and subsequent neurological improvement is provided in Table 3.

A subset of 43 patients with complete datasets was analyzed separately for inferential statistical assessment. Within this cohort, the distribution of preoperative severity followed a similar pattern, with the majority of patients presenting with moderate to severe disease, and no cases classified as mild. This subset was subsequently used to evaluate the statistical association between preoperative severity and postoperative outcomes.

In the subset of 43 patients, the mean preoperative mJOA score was 10.25 (SD 1.80), median 10, mode 12, minimum 7, and maximum 13.

### Functional outcome

Overall, neurological improvement was observed in 166 of 312 patients (53.2%) following surgical decompression. The mean increase in mJOA score was 2.9 points at 6 months postoperatively. Improvement from severe to moderate or mild disability occurred in 91/169 patients (53.8%) with severe CSM, while 75/143 patients (52.4%) with moderate CSM improved to mild disability. A detailed descriptive summary of preoperative severity and postoperative neurological improvement is presented in Table 3.

In the subset of 43 patients, the mean postoperative mJOA score at 6 months was 13.16 (SD 2.43), with a median of 14, a mode of 16, a minimum of 6, and a maximum of 16.

A direct comparison of preoperative and postoperative mJOA scores within the cohort (*n* = 43) is presented in Table 4, demonstrating a clear postoperative improvement.

**Table 3:** Preoperative severity and postoperative neurological improvement

Variable	Value
Severe CSM preoperatively	54.2% (169/312)
Moderate CSM preoperatively	45.8% (143/312)
Mild CSM preoperatively	0%
Severe CSM improved to moderate/mild	53.8% (91/169)
Moderate CSM improved to mild	52.4% (75/143)
Overall neurological improvement	53.2% (166/312)
Mean mJOA improvement	+ 2.9 points

**Table 4:** Shows the comparison of preoperative and postoperative mJOA scores at 6 months in the cohort (*n* = 43), demonstrating a clear improvement in neurological function, with the mean score increasing from 10.25 (SD 1.80) to 13.16 (SD 2.43)

Variable	Preoperative mJOA	Postoperative mJOA
Mean	10.25	13.16
Median	10	14
Mode	12	16
SD	1.80	2.43
Range	6	10
Minimum	7	6
Maximum	13	16

### Adverse events

The overall adverse event rate was 25%. A summary of postoperative complications is provided in Table 5.

**Table 5:** Summary of postoperative complications

Complication category	Specific complication	Number (%)
Wound-related	Superficial wound infection	6 (1.9%)
Wound-related	Wound dehiscence requiring surgical repair	3 (1%)
Neurological	Mild transient neurological deterioration	4 (1.3%)
Neurological	Mild permanent neurological deficit	2 (0.6%)
Neurological	Severe permanent neurological deficit	2 (0.6%)
Urological	Post op urinary retention (Prostatism)	9 (2.9%)
Pulmonary	Postoperative atelectasis	20 (6.4%)
Gastrointestinal	Postoperative dysphagia	13 (4.2%)
ENT	Hoarseness of voice	5 (1.6%)
Surgical	CSF leak	2 (0.6%)
Musculoskeletal	Post-laminectomy neck pain	4 (1.3%)
Neuropsychiatric	Delirium tremens	1 (0.3%)
Neurological	Postoperative C5 palsy	5 (1.6%)
Mortality	Chest infection/ septicemia-related mortality	1 (0.3%)
Mortality	DVT/PE-related mortality	1(0.3%)

### Subset cohort analysis

**Fig. 8:**
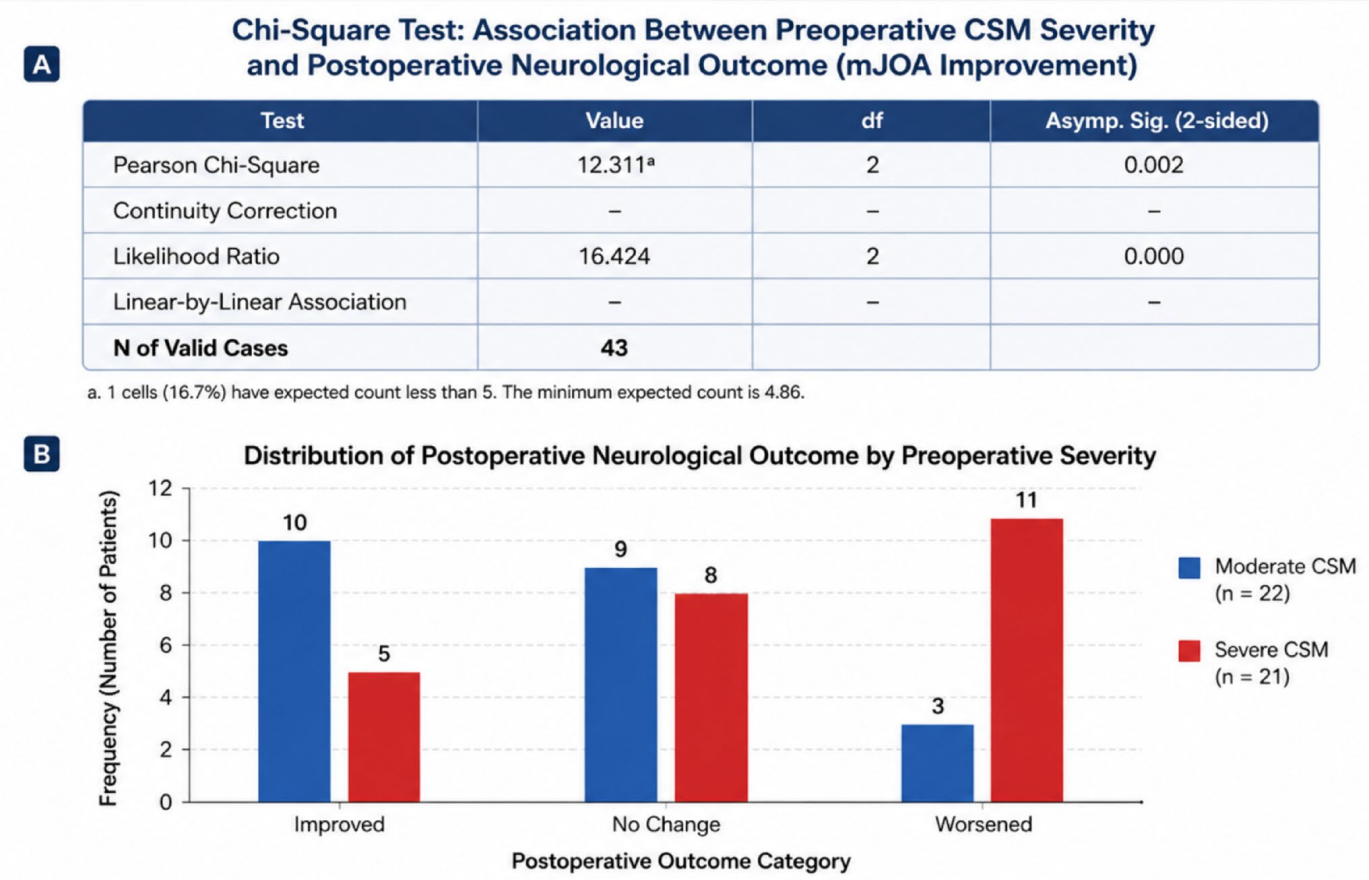
Chi-square analysis of the association between preoperative cervical spondylotic myelopathy (CSM) severity and postoperative neurological outcome in the cohort (*n* = 43). **a** Summary of chi-square test results demonstrating a statistically significant association (Pearson χ² = 12.31, df = 2, *p* = 0.002). **b** Distribution of postoperative outcomes (improved, no change, worsened) stratified by preoperative severity (moderate vs. severe CSM), illustrating the differing outcome patterns between groups

In the subset of 43 patients, inferential statistical analysis using the chi-square test demonstrated a statistically significant association between preoperative CSM severity and postoperative neurological outcome (Pearson χ² = 12.31, df = 2, *p* = 0.002). Patients with severe CSM exhibited a different distribution of postoperative outcomes compared to those with moderate disease, with a higher proportion of unfavorable outcomes. Additionally, patients who underwent anterior cervical decompression demonstrated better neurological outcomes compared to those treated with posterior or combined approaches. These findings were consistent with the descriptive trends observed in the full cohort. An inenicial analysis is shown in Fig. 8.

## Discussion

The findings of this study are consistent with previous literature demonstrating that surgical decompression can lead to meaningful neurological improvement in patients with cervical spondylotic myelopathy, even in the presence of intramedullary signal changes on MRI. In the present study, more than half of the patients showed functional improvement following surgery, with a mean postoperative improvement of 2.9 points in the mJOA score.

The prognostic role of intramedullary signal changes has long been debated. Suri et al. reported that although such signal changes may reflect spinal cord injury, their presence does not necessarily predict poor surgical outcomes [4]. Similarly, Wada et al. found that intramedullary signal changes on MRI may have predictive value but should not be considered absolute indicators of irreversible damage [12]. Histopathological correlation studies have also suggested that MRI signal changes may represent a spectrum of pathological changes ranging from edema to gliosis, which may explain the variability in clinical outcomes [13].

Furthermore, Sarkar et al. demonstrated that T2-weighted intramedullary signal abnormalities can evolve after decompressive surgery, supporting the concept that these changes are not always static markers of permanent injury [9]. In addition, Chen et al. reported in a meta-analysis that certain MRI characteristics may help predict postoperative recovery, although these findings must be interpreted cautiously within the overall clinical and surgical context [3, 14].

Current clinical guidelines emphasize early surgical decompression in patients with degenerative cervical myelopathy to halt disease progression and improve neurological outcomes [10, 15]. Multiple surgical approaches have been described depending on the location and extent of compression. Studies comparing different surgical techniques, including anterior cervical discectomy and fusion and posterior decompressive procedures, found no significant difference between the two approaches. Recent pooled analyses and meta-analyses comparing anterior and posterior surgical approaches for cervical spondylotic myelopathy similarly demonstrated comparable neurological outcomes between operative strategies, with surgical selection primarily guided by the extent and location of compression [16, 17]. Indeed, some authors reported significant neurological improvement following surgical treatment of degenerative cervical myelopathy across different operative strategies [18, 19].

Our findings are also consistent with recent literature highlighting the role of advanced imaging techniques and clinical evaluation in predicting outcomes in patients with degenerative cervical myelopathy [15, 20]. In addition, systematic reviews examining clinical manifestations and functional impairment, such as gait disturbances or tremor, emphasize the heterogeneous presentation of this condition and the importance of early recognition and treatment [18, 21]. In our series, older age, longer symptom duration, and comorbidities were associated with less favorable outcomes, consistent with previously published literature.

The overall adverse event profile observed in this study was likely influenced by delayed presentation, advanced disease severity, and variability in healthcare infrastructure, particularly among patients treated in lower-resource settings. In Sudan, limited access to early diagnosis and specialized spinal care may have contributed to higher complication rates compared with patients treated in more resource-advanced healthcare systems [22]. Nevertheless, despite these challenges, most complications were transient, occurred during the early postoperative period, and improved with appropriate supportive management. These findings further emphasize the importance of early recognition, timely referral, and multidisciplinary perioperative care in optimizing surgical outcomes in cervical spondylotic myelopathy.

The complication profile observed in the present study is comparable to previously published literature on cervical decompression surgery. Tavanaei et al. reported an overall postoperative complication rate of 16%, with common complications including neck swelling, pseudarthrosis, dysphagia, cage/graft subsidence, worsening myelopathy, and hoarseness [23]. Similarly, Badiee et al. reported complication rates ranging from 15 to 25% following posterior cervical decompression and fusion, with both short-term and long-term complications being described [24]. The relatively higher complication rate observed in the present series may partly reflect delayed presentation, advanced disease severity, and variability in healthcare infrastructure across different treatment settings.

Overall, the present findings support the growing body of evidence suggesting that surgical decompression remains an effective treatment for cervical spondylotic myelopathy, even in patients presenting with severe spinal cord compression and intramedullary signal changes on MRI.

This study represents a large, single-surgeon, long-term experience including 312 patients across different healthcare settings, including Sudan, a lower-resource setting. These findings provide valuable real-world insight into surgical outcomes in patients with severe cervical cord compression and intramedullary signal changes, highlighting that the presence of intramedullary signal changes should not be considered a contraindication to surgical intervention, as meaningful neurological recovery can still be achieved.

## Limitations

This study has several limitations. First, its retrospective design introduces the potential for selection bias. Second, the analysis primarily focused on the presence of intramedullary signal changes (IMSCs) without detailed subclassification of signal characteristics (e.g., faint vs. intense T2 changes or T1 hypointensity), partly due to variability in MRI acquisition over the long study period (25 years) and the use of machines with different field strengths (0.5–1.5 Tesla).

Importantly, while descriptive data were available for the full cohort (*n* = 312), complete datasets suitable for inferential statistical analysis were only available for a subset of patients (*n* = 43). As a result, statistical testing was limited to this subgroup, and the remaining cohort was analyzed descriptively. This limitation reflects data availability rather than study design and has been explicitly acknowledged to ensure transparency.

Additionally, potentially relevant confounding variables such as age, duration of symptoms, and comorbidities were not systematically adjusted for due to incomplete data across the full cohort; therefore, residual confounding cannot be excluded.

Finally, the follow-up period of 6–12 months may not fully capture long-term neurological recovery, which can extend beyond one year.

## Conclusion

Surgical decompression is associated with meaningful neurological improvement in patients with cervical spondylotic myelopathy and intramedullary signal changes. Importantly, the presence of IMSCs should not be considered a contraindication to surgery. While descriptive findings demonstrate overall clinical benefit, subset analysis suggests that preoperative severity influences postoperative outcomes. Despite limitations related to study design and imaging variability, these results support early surgical intervention and highlight the need for future prospective studies with standardized imaging and comprehensive multivariable analysis to better define prognostic factors.

## Data Availability

No datasets were generated or analysed during the current study.
